# Severe cardiogenic shock and cardiac arrest due to fulminant cardiac sarcoidosis: a case report

**DOI:** 10.1186/s12872-023-03238-3

**Published:** 2023-05-01

**Authors:** Teja Chakrala, Roshni O. Prakash, Anshul Jain, R. Ashton Vautier, Sahil Prasada, Mohammed Al-Ani, Mustafa M. Ahmed

**Affiliations:** 1 Department of Medicine, University of Florida, 1600 SW Archer Road, Gainesville, FL 32610 USA; 2 Division of Cardiovascular Medicine, University of Florida, Gainesville, FL USA

**Keywords:** Sarcoidosis, Heart failure, Cardiogenic shock, Cardiac arrest, Case report

## Abstract

**Background:**

Cardiac sarcoidosis is found to occur in approximately 5% of patients with sarcoidosis. Its presentation can typically range from complete heart block to ventricular arrhythmias. This condition can rarely present with severe heart failure and cardiogenic shock requiring aggressive and timely management strategies. Advanced imaging techniques are usually required to assist with its diagnosis.

**Case presentation:**

A 70-year-old woman with a history of pulmonary sarcoidosis presented with non-ST elevation myocardial infarction, congestive hepatopathy, and acute renal failure. Left heart catheterization showed evidence of non-obstructive coronary artery disease, and right heart catheterization revealed severely elevated filling pressures and depressed cardiac index. She underwent aggressive diuresis and placement of an intra-aortic balloon pump in addition to initiation of inotropic and vasopressor support. While in the cardiac intensive care unit, she experienced frequent episodes of ventricular tachycardia and went into cardiac arrest requiring cardiopulmonary resuscitation. High clinical suspicion for cardiac sarcoidosis was confirmed by cardiac magnetic resonance imaging findings. After starting immunosuppressive therapy for cardiac sarcoidosis, she demonstrated clinical improvement.

**Conclusion:**

Patients with cardiac sarcoidosis may remain asymptomatic or present with conduction abnormalities and arrhythmias. They rarely present with severe biventricular heart failure and cardiogenic shock, and in such cases, they require timely initiation of pharmacologic and device therapies, along with implementation of mechanical circulatory support.

**Supplementary Information:**

The online version contains supplementary material available at 10.1186/s12872-023-03238-3.

## Background

Cardiac sarcoidosis (CS) is an uncommon condition found to occur in a fraction of patients with sarcoidosis [1]. It is associated with interstitial myocardial fibrosis and scarring, leading to damage of the cardiac conduction system [2]. CS can remain asymptomatic or present as clinically significant heart failure, heart block, or ventricular arrhythmias [2, 3]. Severe heart failure and cardiogenic shock due to fulminant cardiac sarcoidosis is rare and requires a multimodal management approach with inotropes, vasopressors, immunosuppressive, and anti-arrhythmic agents. Close clinical monitoring is crucial to ensure timely recovery [4].

## Case presentation

### Days 1 and 2 (6/11/22 – 6/12/22)

**Fig. 1:**
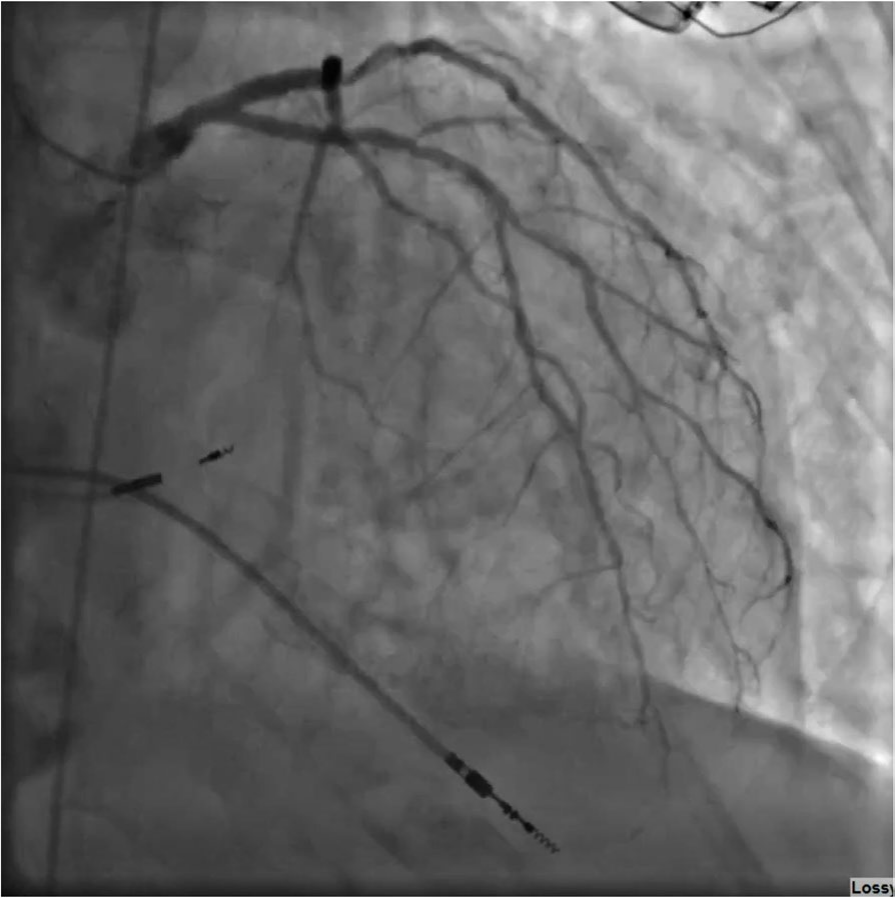
Coronary angiography showing moderate non-obstructive disease. (Video File)

A 70-year-old woman with a past medical history of pulmonary sarcoidosis and high-degree atrioventricular (AV) block status-post pacemaker implantation presented to a nearby hospital for persistent chest pain, nausea, and dizziness. On presentation, she was tachycardic to 103 beats per minute (bpm) with a blood pressure of 93/55 mmHg. Initial labs showed a white blood cell count (WBC) of 15,900/mm3 and platelets of 435,000/uL; complete metabolic profile (CMP) demonstrated a sodium of 125 mEq/L, creatinine 0.9 mg/dL, aspartate aminotransferase (AST) of 332 IU/L, and alanine aminotransferase (ALT) of 346 IU/L. Peak high-sensitivity troponin was 9,180 pg/ml and N-terminal pro b-type natriuretic peptide (NT-proBNP)was 30,919 pg/ml (normal < 100 pg/mL). She was diagnosed with non-ST elevation myocardial infarction due to her presenting symptoms and rise in troponin levels. Left heart catheterization (LHC) showed non-obstructive coronary artery disease (CAD) (Fig. 1), with a left ventricular end-diastolic pressure (LVEDP) of 35 mmHg. TTE showed an ejection fraction of 30–35%, left ventricular septal wall akinesis, moderate tricuspid and mitral regurgitation, and mild concentric left ventricular hypertrophy. The patient was commenced on intravenous (IV) diuresis and goal-directed medical therapy for decompensated heart failure. She quickly decompensated and developed increasing pressor and inotropic agent requirements in the setting of multiorgan failure. Transfer to our institution for further evaluation and management was initiated.

### Days 3 and 4 (6/13/22 – 6/14/22)

**Fig. 2:**
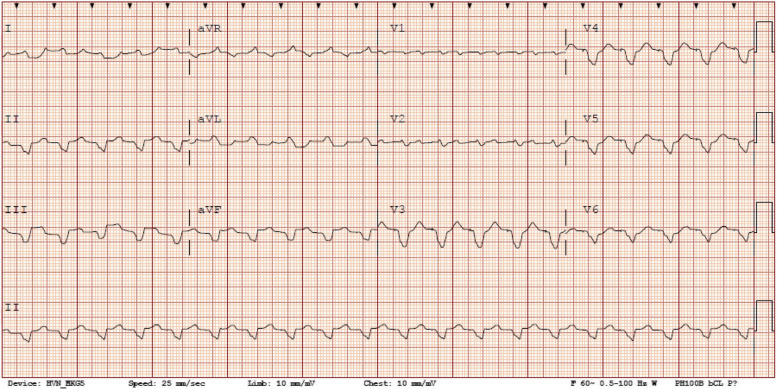
Admission ECG showing ventricular paced rhythm at 119 bpm

Upon transfer, the patient was admitted to our cardiac intensive care unit for cardiogenic shock. On arrival, she had a blood pressure of 85/52 mmHg, heart rate of 119 bpm, temperature of 36.4℃ (97.5℉), respiratory rate of 29 breaths/minute and oxygen saturation of 95% on room air. Her physical exam was notable for cool distal extremities and weak peripheral pulses. Labs revealed a WBC count of 20,900/mm3 and CMP showed a sodium of 122 mEq/L, creatinine of 1.85 mg/dL (baseline 0.75 mg/dL), AST 2,394 IU/L, ALT 3,314 IU/L. Lactic acid was 3.9 mmol/L and b-type natriuretic peptide (BNP) was > 4,900 pg/mL (NT-proBNP was not performed at our institution) (Table 1). Electrocardiogram (ECG) obtained on admission is shown in Fig. 2. The differential diagnosis for this patient’s biventricular heart failure leading to cardiogenic shock included CS, giant cell myocarditis, infectious myocarditis, stress cardiomyopathy, and other infiltrative cardiomyopathies. TTE at our facility showed severe biventricular systolic dysfunction with a left ventricular ejection fraction (LVEF) of 20–25% with akinesis of the basal, mid-inferior, and septal walls in addition to moderate tricuspid and mitral valve regurgitation. The patient was started on IV dobutamine and IV norepinephrine for management of cardiogenic shock at the referring institution, which were continued upon arrival. She was subsequently taken to the cardiac catheterization laboratory for RHC. Right heart catheterization (RHC) demonstrated severely elevated biventricular filling pressures with a depressed cardiac index of 1.2 L/min/m^2^ (Table 2). Pulmonary artery pulsatility index was reduced to 0.56, suggestive of concomitant right ventricular dysfunction. Mechanical circulatory support was provided via right femoral intra-aortic balloon pump (IABP) placement with anticoagulation achieved with IV heparin.

**Table 1:** Table of lab values obtained at our institution upon transfer and discharge

**Laboratory** **Result**	**Reference** **Range**	**Admission to** **CCU** **6/13**	**Discharge** **7/7**
Liver Function Tests (LFT)			
AST, IU/L	0 – 37	2,394	17
ALT, IU/L	0 – 35	3,313	36
ALP, IU/L	33 – 133	210	78
Basic Metabolic Panel (BMP)			
Sodium, mmol/L	136 – 145	122	135
Potassium, mmol/L	3.3 – 5.1	4.3	4.1
Carbon dioxide, mmol/L	22 – 30	15	26
Urea Nitrogen, mg/dL	6 – 21	36	25
Creatinine, mg/dL	0.38 – 1.02	1.97	0.68
Other			
Lactic Acid, whole blood, mmol/L	0.3 – 1.5	3.9	N/A
White blood cell count, 1000/mm^3^	4.0 – 10.0	20.9	10.7
BNP, pg/mL	< 100	> 4,900	N/A

**Table 2:** Invasive Hemodynamics

	**RAP** **(mmHg)**	**PAP** **(mmHg)**	**PCWP** **(mmHg)**	**CO/CI** **(Fick)**
Admission	18	35/25 (mean 26)	25	2.4/1.3
IABP removal	13	35/22 (mean 29)	25	3.4/1.8

*RAP* Right arterial pressure, *PAP* pulmonary arterial pressure, *PCWP* pulmonary capillary wedge pressure, *CO/CI* cardiac output/cardiac index

### Days 5 – 14 (6/15/22 – 6/29/22)

**Fig. 3:**
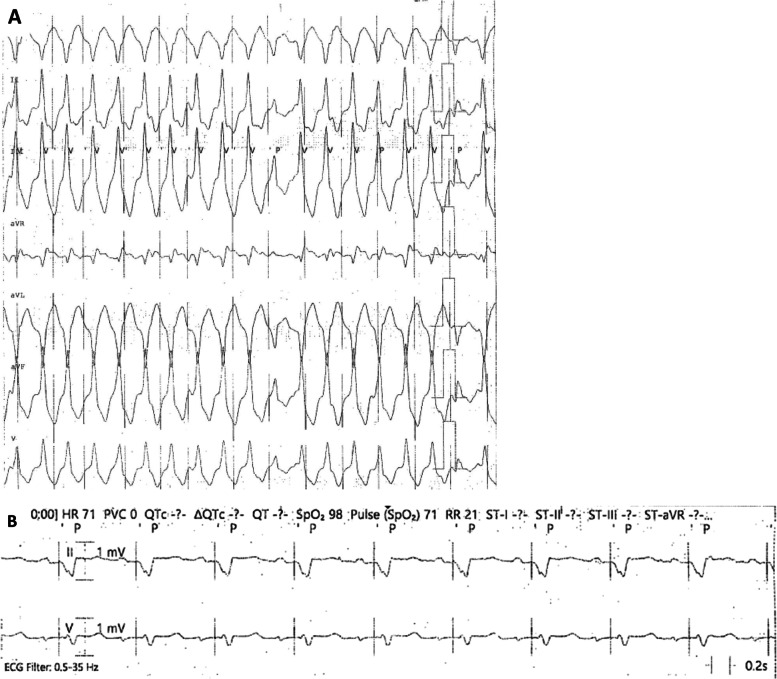
Telemetry recordings. **A** Monomorphic ventricular tachycardia **B** Ventricular-paced rhythm prior to episode

**Fig. 4:**
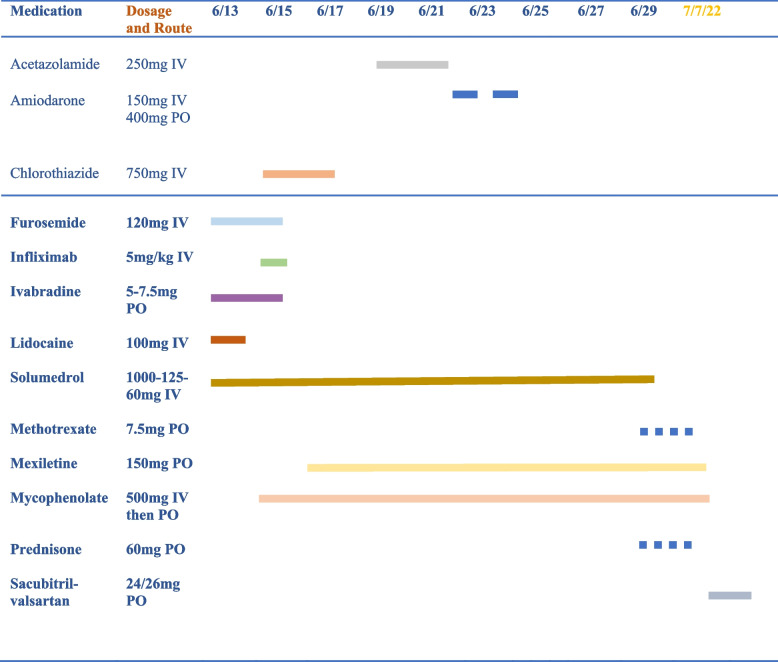
Therapies Used in Management by Date

Two days after IABP placement, our patient experienced ventricular tachycardia (VT) arrest with return of spontaneous circulation achieved after one round of cardiopulmonary resuscitation and defibrillation. She converted to normal sinus rhythm and was started on IV amiodarone and IV lidocaine infusions (Figure 3a. Baseline rhythm prior to VT episode seen in Fig. 3b). Due to her significant transaminitis and concern for structural heart disease, she was subsequently transitioned to oral mexiletine. She was started on intense medical management for refractory volume overload with continuation of IV furosemide and initiation of IV chlorothiazide. Ivabradine was added at 5 mg and increased to 7.5 mg twice daily. A short-term dialysis catheter was placed in anticipation of possible ultrafiltration requirements given her initial anuria, however this soon resolved following optimized diuretic dosing. IV acetazolamide was required briefly due to metabolic alkalosis. The patient demonstrated hemodynamic improvement with this multimodal approach and was eventually able to be weaned off inotropic and vasopressor support (timeline of administered medications is demonstrated in Fig. 4). However, when attempting to wean her balloon pump, she experienced frequent episodes of VT requiring several IV boluses of amiodarone. Eventually, she was weaned off IABP support successfully.

### Days 15 – 18 (6/30/22 – 7/3/22)

**Fig. 5:**
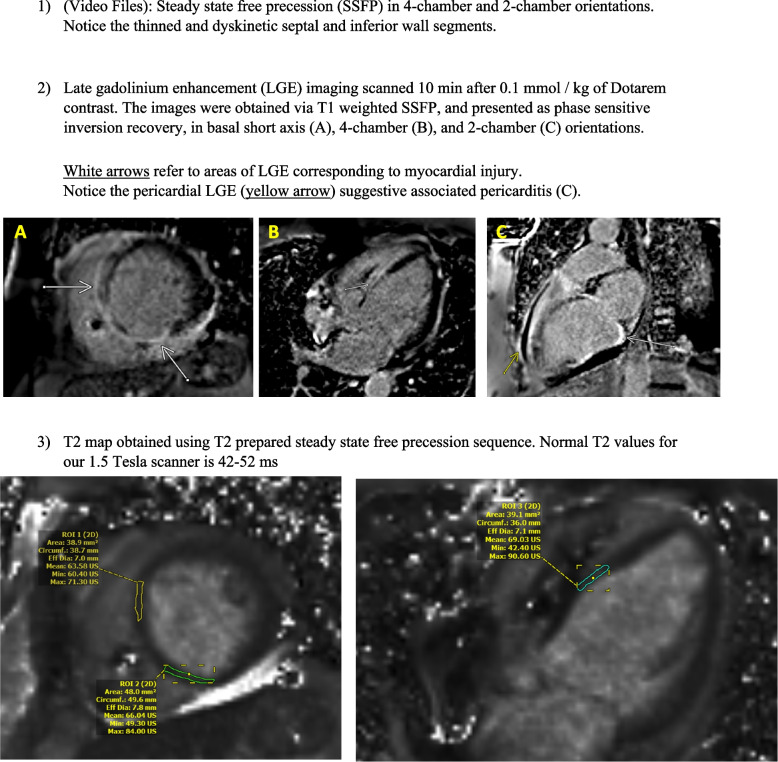
Cardiac magnetic resonance imaging findings. 1) (Video Files): Steady state free precession (SSFP) in 4-chamber and 2-chamber orientations. Notice the thinned and dyskinetic septal and inferior wall segments. 2) Late gadolinium enhancement (LGE) imaging scanned 10 min after 0.1 mmol / kg of Dotarem contrast. The images were obtained via T1 weighted SSFP, and presented as phase sensitive inversion recovery, in basal short axis (**A**), 4-chamber (**B**), and 2-chamber (**C**) orientations. White arrows refer to areas of LGE corresponding to myocardial injury. Notice the pericardial LGE (yellow arrow) suggestive associated pericarditis (**C**). 3) T2 map obtained using T2 prepared steady state free precession sequence. Normal T2 values for our 1.5 Tesla scanner is 42–52 ms

**Fig. 6:**
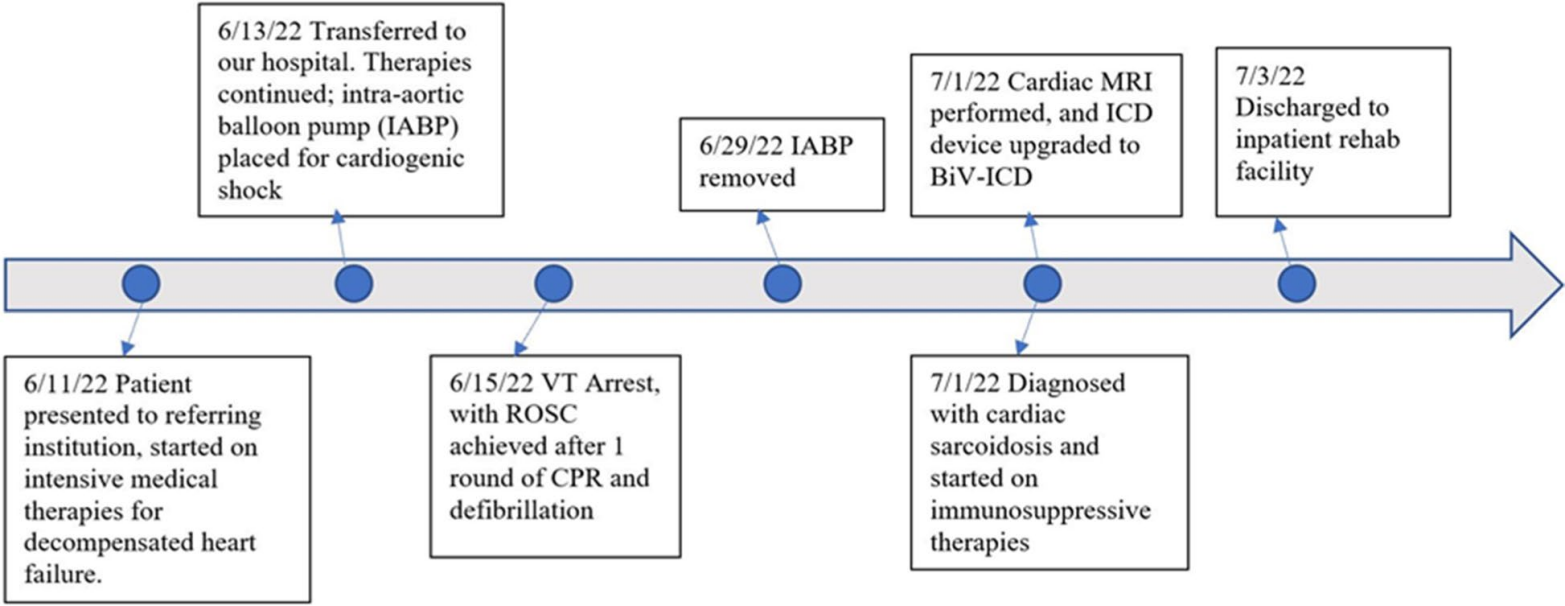
Timeline of events

At this stage, suspicion for cardiac sarcoidosis heightened given the patient’s frequent VT episodes, presence of advanced AV block, and history of biopsy-proven pulmonary sarcoidosis. To rule out other possible cardiomyopathies as an etiology for her cardiogenic shock, cardiac magnetic resonance imaging (CMR) was performed. It showed significant late gadolinium enhancement (LGE), T1 and T2 prolongation in the septum, inferior, and lateral walls, in addition to associated pericardial LGE, indicative of severe myopericarditis (Fig. 5). With the support of suggestive findings on CMR, we diagnosed our patient with cardiac sarcoidosis. Other underlying causes of her decompensation had been reasonably excluded. As a result, she was started on immunosuppressive therapy with IV pulse dose steroids followed by oral steroids, to which she had responded well. She was initiated on mycophenolate mofetil at 1000 mg twice daily and infliximab 10 mg/kg. Afterwards, she continued to demonstrate clinical improvement with end-organ recovery. She underwent replacement of her existing pacemaker device to a biventricular implantable cardioverter-defibrillator given her history of sustained VT and chronic RV pacing, shortly after the CMR. She was discharged to an inpatient rehabilitation facility on sacubitril/valsartan, metoprolol, mycophenolate, and oral prednisone. Figure 6 displays a timeline of major events during this hospitalization.

The patient was seen in our Heart Failure Clinic two weeks after discharge and was noted to be euvolemic and making progress in her rehabilitation.

## Discussion

CS is estimated to occur in approximately 5% of patients with sarcoidosis with this number rising over the recent years. This increase is likely attributed to an improvement in diagnostic modalities rather than an actual increase in disease prevalence [1]. Clinical presentations tend to be heterogenous, ranging from heart failure, ventricular arrythmias, heart block, and sudden cardiac death, with the extent of left ventricular dysfunction hypothesized as being the most important prognostic indicator of disease mortality [2, 3]. CS is inherently associated with inflammation of the myocardium along with interstitial fibrosis, inadvertently leading to nodal conduction slowing and development of re-entrant arrythmias, as seen in patients similar to ours [4].

Establishing a diagnosis of CS tends to be difficult in part due to the lack of specific imaging patterns that are pathognomonic of the condition [1, 3]. Patients typically exhibit conduction abnormalities on ECG, with varying degrees of AV nodal and bundle branch blockade as the most reported findings in studied CS cohorts [1]. Findings on TTE are often variable, however certain features such as basal interventricular thinning, as seen in our patient, have been frequently seen in patients with CS [3]. Other reported findings found on TTE in these patients include left ventricular and right ventricular systolic dysfunction, along with wall motion abnormalities in a non-coronary distribution.

CMR showing LGE in the basal segments, especially the septum and lateral wall, is commonly seen in CS [3, 5]. No specific pattern of LGE however is diagnostic of CS, although it is typically reported to be multi-focal and patchy with endocardial sparing [3]. Additionally, this study was performed in our patient despite having abandoned leads and CIED device on the left chest, which are not true contraindications to CMR. Diagnostic images may still be obtained with appropriate acquisition techniques. Fluorodeoxyglucose positron emission tomography (FDG-PET) is also being increasingly utilized for diagnosing patients with CS and has been reported to be a useful disease activity marker for guiding therapy in these patients [5, 6]. Patterns of focal, diffuse, and focal-on-diffuse uptake are typically seen in patients with active CS. Both CMR and FDG-PET are highly sensitive imaging modalities for identifying CS, however the latter tends to be more sensitive in detecting acute active disease when compared to T2 mapping (which images edema). Endomyocardial biopsy (EMB), when pursued, should be guided either by electroanatomic mapping or image guidance via CMR or FDG-PET [1, 6]. This is due to the focal nature of the disease as evidenced by the significant increase in diagnostic sensitivity of EMB when utilizing these modalities for guidance. Novel techniques including measurement of serum levels of biomarkers such as neopterin and interleukin-2 receptor have been shown to be significantly elevated in patients with active CS, however further research is required prior to implementation in clinical practice [1, 3].

Currently a diagnosis of CS is established based on the 2014 Expert Consensus Guidelines put forth by the Heart Rhythm Society and several other organizations [6]. Per these guidelines, a diagnosis of CS can be made by the following 2 pathways:

1. Histological diagnosis from myocardial tissue: CS is diagnosed in the presence of non-caseating granuloma on histological examination of myocardial tissue with no alternative cause identified (including negative organismal stains if applicable).
2. Clinical Diagnosis from Invasive and Non-Invasive Studies: It is probable (where ‘probable involvement’ is considered adequate to establish a clinical diagnosis of CS) that there is CS if:
  A. There is a histological diagnosis of extra-cardiac sarcoidosis, *and* 
  B. One or more of following is present:
    1. Steroid ± immunosuppressant responsive cardiomyopathy or heart block
    2. Unexplained reduced LVEF (< 40%)
    3. Unexplained sustained (spontaneous or induced) VT
    4. Mobitz type II 2nd degree heart block or 3rd degree heart block
    5. Patchy uptake on dedicated cardiac PET (in a pattern consistent with CS)
    6. LGE on CMR (in a pattern consistent with CS)
    7. Positive gallium uptake (in a pattern consistent with CS), *and* 
  C. Other causes for the cardiac manifestation(s) have been reasonably excluded.

Management of patients with CS involves a combination of pharmacologic and device therapies [5, 6]. Immunosuppression should be considered in patients with evidence of myocardial inflammation along with findings of heart block, ventricular ectopy, sustained ventricular arrhythmias, and/or left ventricular dysfunction [3, 5]. Corticosteroids are typically utilized as a first line agent, with a recommended initial dose of 30 to 40 mg/day. Kandolin et. al. in a study of 110 patients with CS showed that corticosteroid therapy resulted in an improvement of left ventricular function in patients with a severely impaired LVEF < 35% [7]. Kumar et. al. demonstrated that corticosteroids may be effective for CS complicated by ventricular arrhythmias in patients with less advanced left ventricular dysfunction with a significant decrease in the number of premature ventricular contractions and non-sinus tachycardia prevalence [4]. Other medications that may be utilized as second- and third-line agents include methotrexate, azathioprine, cyclophosphamide, and infliximab [4]. CS-related malignant arrhythmias such as VT storm are responsive to short courses (3 to 5 days) of IV corticosteroid therapy in addition to anti-arrhythmic medications [8]. Catheter ablation, particularly in the inflammatory phase, is associated with a high degree of failure and high rates of VT recurrence [4, 8]. Orthotopic heart transplant may be indicated in severe cases of CS unresponsive to drug and device therapy [5, 8].

This case report contributes to the growing body of knowledge with regards to severe presentations of CS and close clinical monitoring required in its care. Limitations of our report include its rare presentation, making it difficult to identify in clinical practice along with EMB not having been performed on our patient.

## Conclusion

Cardiac sarcoidosis rarely presents as cardiogenic shock. This case highlights the importance of including CS in the differential diagnosis of patients with refractory heart failure along with the complex treatment options that can be utilized during such severe presentations. Advanced cardiac imaging and EMB, when indicated, can assist in establishing a diagnosis.

## Supplementary Information

Additional file 1.

Additional file 2.

## Data Availability

Data including lab values, imaging files and other raw data was obtained from the patient’s medical record and can be made available in a de-identified format.

## Abbreviations

LHC: Left heart catheterization
TTE: Transthoracic echocardiogram
AV: Atrioventricular
CS: Cardiac sarcoidosis
IABP: Intra-aortic balloon pump
IV: Intravenous
LGE: Late gadolinium enhancement
CMP: Complete metabolic profile
ALT: Alanine aminotransferase
AST: Aspartate aminotransferase
CAD: Coronary artery disease
BNP: B-type natriuretic peptide
LVEF: Left ventricular ejection fraction
VT: Ventricular tachycardia
CMR: Cardiac magnetic resonance imaging
EMB: Endomyocardial biopsy
ECG: Electrocardiogram
